# NAVIGATING AGING ACROSS CULTURES: THE FORTITUDE IN PERSPECTIVES FROM KOREANS AND BEYOND

**DOI:** 10.1093/geroni/igae098.1667

**Published:** 2024-12-31

**Authors:** Michin Hong, Kyungmin Kim

**Affiliations:** Chair; Discussant

## Abstract

The symposium aims to provide a multifaceted examination about aging related issues within different cultural contexts with a particular focus on the Korean population. The first study conducted a cross-cultural examination on financial satisfaction during the retirement transition period between South Kore and the U.S., highlighting the needs for culturally tailored interventions to support individuals’ financial wellbeing in retirement. The second study focused on Korean middle-aged adults’ residential preferences after retirement, emphasizing the importance of understanding evolving housing needs and their impacts on health and social interactions in later life. Third study conducted a comparative analysis of long-term care policies between South Korea and Singapore, examining the emergence of Community Care services as a potential solution for aging-in-place and providing valuable insights into the compatibility of such services with different cultural and welfare system framework. The last study introduced a psychometrically sound scale to measure public stigma associated with Alzheimer’s disease among Korean-speaking populations, contributing to a better understanding of the nature and magnitude of Alzheimer’s disease stigma, with implications for stigma reduction efforts within this population. The symposium offers an excellent opportunity to examine various aspect of aging issues among Koreans, encompassing financial wellbeing, housing preference, long-term care, and public perception of an age-related disease. By examining these topics, the symposium will contribute to informing policy and practice in addressing various needs of aging populations from a more culturally tailored perspective. Korean/Korean American and Aging Interest Group Sponsored Symposium

